# Artificial intelligence-based pulmonary embolism classification: Development and validation using real-world data

**DOI:** 10.1371/journal.pone.0305839

**Published:** 2024-08-21

**Authors:** Luan Oliveira da Silva, Maria Carolina Bueno da Silva, Guilherme Alberto Sousa Ribeiro, Thiago Fellipe Ortiz de Camargo, Paulo Victor dos Santos, Giovanna de Souza Mendes, Joselisa Peres Queiroz de Paiva, Anderson da Silva Soares, Márcio Rodrigues da Cunha Reis, Rafael Maffei Loureiro, Wesley Pacheco Calixto

**Affiliations:** 1 Department of Radiology, Hospital Israelita Albert Einstein, Sao Paulo, Brazil; 2 Electrical, Mechanical & Computer Engineering School, Federal University of Goias, Goiania, Brazil; 3 Institute of Informatics (INF), Federal University of Goias, Goiania, Brazil; 4 Technology Research and Development Center (GCITE), Federal Institute of Goias, Goias, Brazil; University of Manitoba, CANADA

## Abstract

This paper presents an artificial intelligence-based classification model for the detection of pulmonary embolism in computed tomography angiography. The proposed model, developed from public data and validated on a large dataset from a tertiary hospital, uses a two-dimensional approach that integrates temporal series to classify each slice of the examination and make predictions at both slice and examination levels. The training process consists of two stages: first using a convolutional neural network InceptionResNet V_2_ and then a recurrent neural network long short-term memory model. This approach achieved an accuracy of 93% at the slice level and 77% at the examination level. External validation using a hospital dataset resulted in a precision of 86% for positive pulmonary embolism cases and 69% for negative pulmonary embolism cases. Notably, the model excels in excluding pulmonary embolism, achieving a precision of 73% and a recall of 82%, emphasizing its clinical value in reducing unnecessary interventions. In addition, the diverse demographic distribution in the validation dataset strengthens the model’s generalizability. Overall, this model offers promising potential for accurate detection and exclusion of pulmonary embolism, potentially streamlining diagnosis and improving patient outcomes.

## Introduction

Pulmonary embolism (PE) is a vascular occlusion occurring within the pulmonary arterial system, most commonly caused by a thromboembolus originating from the deep venous system (usually the legs). This blockage disrupts blood flow and gas exchange within the lungs, leading to various clinical presentations and potentially life-threatening complications. [1]. The diagnosis of PE can be challenging due to the variety of clinical manifestations and the overlap of symptoms with other diseases [2]. Therefore, rapid and accurate identification is essential [3–5]. PE poses a significant public health threat, affecting 1-2 people per 1,000 annually [6]. It is the third leading cause of cardiovascular death after stroke and myocardial infarction [7], with mortality rates in the US alarmingly on the rise over the past decade [4].

Common clinical symptoms of PE include dyspnea, pleuritic chest pain, cough, substernal chest pain, hemoptysis, and syncope. The variety of clinical presentations, observed in many other pulmonary disorders, plus the overlap of symptoms often lead to delayed diagnosis, emphasizing the need for high vigilance by specialists to recognize it in time [4]. Computed tomography pulmonary angiography (CTPA) remains the standard method for PE diagnosis given its safety, speed, and accuracy. However, manual analysis of CTPA images is time-consuming (approximately 20 minutes per scan) and prone to error due to radiologist fatigue and varying levels of experience [1, 2]. In addition, CTPA allows risk stratification by detecting dilatation of the right ventricle, increase in the caliber of the pulmonary artery trunk, rectification of the interatrial septum and inversion of the RV/LV ratio, which is a predictor of mortality [3]. Despite its importance, underdiagnosis of PE remains a major problem, particularly in developing countries. For example, while the annual rate of PE hospitalizations in Brazil was 4.7/100,000 population, the United States had a PE hospitalization rate of 112.3/100,000 population. This indicates a high number of underestimated cases, which is particularly undesirable as the prognosis of the disease is highly dependent on appropriate and timely diagnosis [8].

The application of artificial intelligence (AI) techniques, such as machine learning and medical image processing, stands out in several medical specialties. Since the 2010s, there have been advances in AI, particularly in the field of deep learning (DL), which is revolutionizing the handling of complex medical data [2]. The ability of AI models to process large amounts of information and recognize subtle patterns shows significant potential for improving medical diagnoses. Before the 2010s, in the 1990s, AI models worked on problems of assisted interpretation of perfusion lung scintigraphy for the diagnosis of artificial PE [9] and the clinical diagnosis of acute PE [10]. In 2018, the use of AI models has emerged as a promising approach to improve the accuracy and efficiency of the classification of exams related to PE [3, 11–14]. In particular, the application of AI models in the classification of imaging exams, such as chest computed tomography (CT) and lung scintigraphy, is characterized by their ability to assist physicians in detecting signs of PE [2].

In the study conducted by Cano-Espinosa *et al.* [15], three variants of the U-net segmentation network are tested: with 2D convolutions, with 2D convolutions and 3D input images, and with 3D convolutions. The most promising result was achieved by the network with 3D convolutions, in which the network output value in the most central voxel of each PE is used to determine whether it is a true positive or not. The authors obtained a recall of 55% and a rate of 1 false positive per second (FP/s). The limitation of this study is related to the database, which included only 60 computed tomography (CT) exams. Rajan *et al.* [16] implemented the U-net network with 2D convolutions, using the neighborhood of the slices as different input channels. This strategy expands the context and allows the 2D U-net to generate high-quality masks for the detection step. The authors achieved an ROC curve of 0.85 on the test set and 0.94 on the validation set. The limitation of this work relates to the validation of the model against an external database. Huang *et al.* [17] developed the PENet network based on DenseNet and U-net to classify exams as positive or negative for PE. The method was pre-trained using transfer learning, resulting in a ROC curve of 0.85. The limitation of the work lies in the ability of the model to recognize only features related to PE.

Tajbakhsh *et al.* [18] proposed a method based on the location and segmentation of PE candidates, as well as the reduction of false positives through the use of U-net and connected components labeling algorithm. The aim was to find PE candidates in three databases, resulting in a recall of 0.89. The limitation of the work is related to the number of false negatives, elements in the image that indicate the presence of PE, but are not identified by the model. The research conducted by Kiourt *et al.* [19] proposed a method for localizing and classifying PE using deep learning algorithms. Eight convolutional neural network architectures were tested to find the best-performing architecture for the problem, in addition to the You Only Look Once v4 (YOLOv4) algorithm to identify the region of interest (RoI). An accuracy of 91.63% was achieved. The limitation of the work is related to the database, as the computed tomography exams were performed with a single scanner, which affects the quality compared to studies with images from different scanners.

Huhtanen *et al.* [20] developed and evaluated a deep neural network model for PE detection using CTPA. The model is divided into two parts: i) using a convolutional neural network that combines concepts from the Inception architecture with ideas from the residual neural network (ResNet), called InceptionResNet V_2_, and ii) recurrent neural network designed to address the challenge of retaining important information over long time periods (long short-term memory, LSTM). Both are used in the processing of CTPA stacks, which are treated as slice sequences. The data has no annotation or segmentation, only binary labels indicating positive or negative for PE in each slice with a thickness of 3*mm*. Transfer learning was applied in both models. The InceptionResNet V_2_ network was pre-trained with the National Institutes of Health chest X-ray dataset, while the LSTM network was pre-trained with ImageNet, the visual dataset used for training and evaluating image recognition algorithms. The most promising results were obtained with the InceptionResNet V_2_ network: an ROC curve of 0.94, sensitivity of 86.6%, and specificity of 93.5% in predicting PE for CTPA stacks. However, the study lacked validation on external data and did not present the loss curve for internal validation. Several recent studies have explored the potential of AI models in PE detection, each with different approaches and specific challenges, as summarized in Table 1.

**Table 1:** Some studies related to the classification and segmentation of pulmonary embolism.

Method	References	Description
Segmentation	[15]	PE segmentation technique with 2D/3D architecture
Segmentation	[16]	2D segmentation technique with context-augmentation
Classification	[17]	Classification technique with transfer learning
Segmentation	[18]	Candidate PE detection and segmentation technique
Detection and classification	[19]	Benchmark of convolutional neural network architectures for PE localization and classification
Classification	[20]	Slice- and exam-level PE classification model using binary labels

There is an important gap in the research of PE detection models: the lack of validation across diverse datasets. Many studies rely on limited or device-specific data, raising concerns about their generalizability to clinical settings. In addition, research tailored to large public health systems is scarce. This lack of diverse validation datasets and real-world context limits our understanding of how well the models perform across variations in data such as slice thickness, demographics, and image sources. Addressing these issues is critical to developing reliable PE detection models that can be used effectively in different healthcare settings. The presentation of these perceptions justifies this work.

In the proposal of this work, the originality of the hybrid model stands out, which is composed of the convolutional neural network architecture for feature extraction and a recurrent neural network for classification at the image and exam level (CNN-LSTM), using the Universal Repository for medical images. The model is trained with a public dataset and then validated with an external dataset covering different slice thicknesses and showing variability in terms of gender and age. This leads to the primary hypothesis: if the CNN-LSTM hybrid model is trained with a public dataset and evaluated with an external dataset that contains variations in slice thicknesses of CT images as well as a wide range of demographic diversity, then it can perform adequately and consistently in classifying PE in different clinical contexts. The development of a model trained with data from countries with continental coverage, combined with validation by board-certified thoracic radiologists, is relevant for progress in the medical field. The deep learning approach to detecting and classifying PE, together with research into new forms of mixed architectures, represents a major challenge.

The main objective of this study is to develop a deep neural network capable of classifying PE in CTPA images at the slice and volume level using the Universal Repository of medical images for validation. Specific objectives include: i) implementation, integration, and addition of new architectures, ii) training with a public health database, and iii) validation of the model with databases containing exams from different devices. The research is organized for clear comprehension. The “Theoretical Foundation” establishes the core concepts for understanding the subsequent methodology and results. The “Methodology” then details the proposed development, training, and validation processes for the deep neural network. The “Results” section presents and describes the findings obtained from applying this approach. Following this, the “Discussion” analyzes and interprets these findings, exploring their implications and limitations. Finally, the research culminates in a “Conclusion” that summarizes the key takeaways and explores potential future research directions.

## Theoretical background

This section introduces concepts related to CTPA, protocols for CTPA, machine learning on medical images, and preprocessing of CT images. These concepts form the basis for understanding the proposed methodology.

### Computed tomography pulmonary angiography

**Fig 1:**
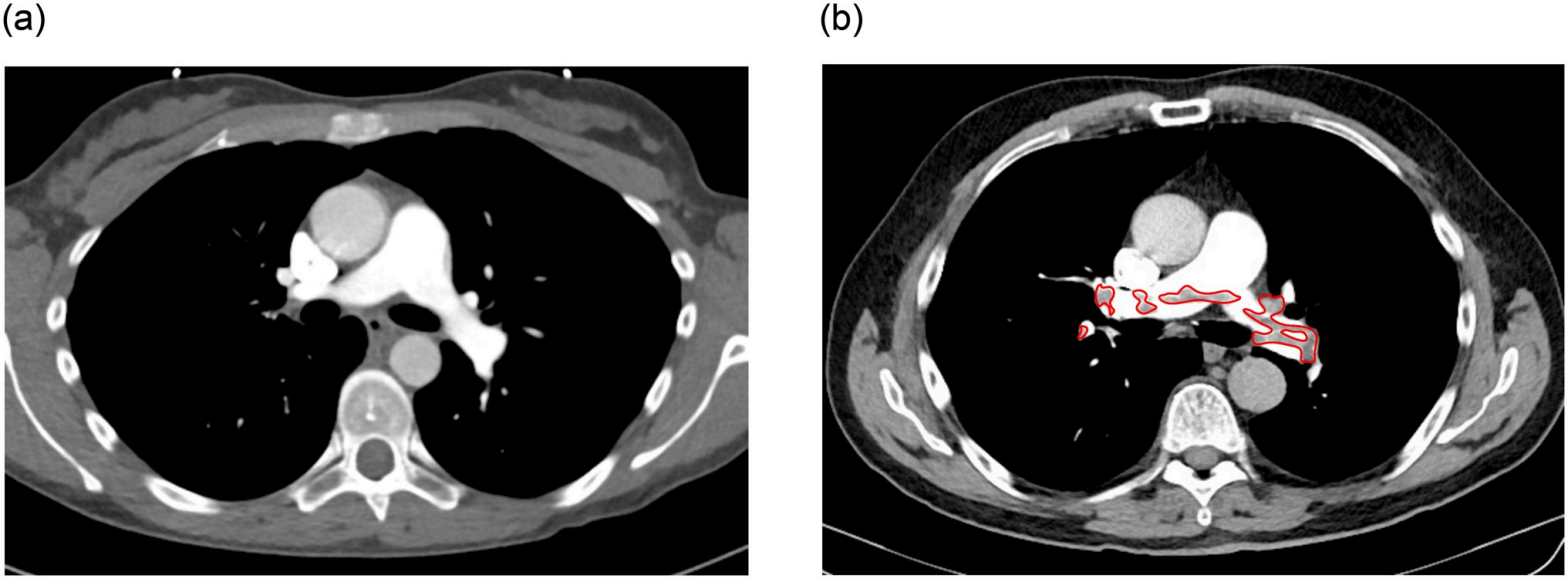
Illustration of CT pulmonary angiography exams: (a) axial C+ classified as normal and (b) axial C+ classified with the presence of PE indicated in the markings.

CTPA is the standard imaging technique for the diagnosis of PE, which is based on the detection of filling defects in the pulmonary arterial system. Slightly different protocols are used in different radiologic settings to achieve the same goal: diagnostic contrast filling of the pulmonary arteries and their branches [21]. Late acquisition may make it difficult to differentiate between arterial and venous pulmonary branches. Fig 1(a), taken from Hacking [22] and Fig 1(b), adapted from Knipe [23], illustrate two examples of CTPA, one considered normal and another featuring PE, respectively.

In the title of Fig 1, the term axial C+ specifies that the CTPA examination was conducted in cross-sectional cuts of the lungs with the application of a contrast agent. Fig 1(b) illustrates filling defects observed in the trunk of the pulmonary artery, as well as in the right and left branches. The CTPA is a rapid, widely available, and minimally invasive imaging technique that offers several advantages in the diagnosis and treatment of lung diseases. It enables the detection of abnormalities within the pulmonary arteries, including pulmonary emboli, aneurysms, stenoses, or malformations. This technique also diagnoses a wide range of lung diseases such as tumors, infections, and inflammation. In addition, CTPA provides important information for procedures such as lung biopsies and vascular interventions. It also helps to monitor the effectiveness of treatment and detect respiratory diseases. Finally, CTPA provides a detailed 3D view of the lungs and blood vessels, which is invaluable for surgical planning. [24–27].

While CTPA proves a valuable tool, its application comes with inherent challenges and considerations. Radiation exposure remains a concern despite the use of low-dose techniques [25, 28]. Patients with iodine contrast allergies face heightened risks of adverse reactions, including anaphylaxis [29, 30]. In patients with renal insufficiency, strategies such as prior assessment of renal function and the correct choice of contrast agent are crucial [31, 32]. In addition, certain medical conditions and physical limitations may preclude the use of CTPA. These include pregnancy, severe claustrophobia, or difficulty holding a certain position during the scan [33, 34]. In patients with acute thyroid storm, iodinated contrast medium exposure can potentiate thyrotoxicosis; in such patients, iodinated contrast medium should be avoided [35]. The financial burden of the procedure may also be an obstacle for some patients or healthcare systems. Moreover, image interpretation requires a high level of expertise, as errors can lead to misdiagnosis or overlooked important findings. Therefore, careful consideration of the benefits and risks for each patient and clinical situation is essential [36, 37].

### Protocols for pulmonary CT angiography imaging

The specific protocols for CTPA imaging may vary depending on the medical facility, but generally follow some common guidelines [38]. The low-dose protocol is particularly considered for more sensitive patients, such as pregnant women and children. The standard protocol provides for the acquisition of images at different phases of the cardiac cycle to optimize visualization of the pulmonary arteries. The contrast agent protocol adapted to the cardiac phase correlates with the circulation time of the contrast agent. The high-resolution protocol is used for detailed assessment of lung structures and is useful to detect small lung lesions, tumors, or other diseases [39]. The protocol for patients with renal insufficiency carefully considers the administration of contrast media and adjusts the dose according to the patient’s renal function [40]. The choice of protocol depends on the clinical suspicion, the characteristics of the patient, and the technology available at the radiology center. Each protocol aims to optimize image quality, minimize risks, and adapt to the specific needs of each situation [41].

The protocols used in CTPA should provide high-quality images necessary for medical analysis. The images must highlight the pulmonary arteries in relation to the surrounding tissues and provide enough spatial resolution to reveal anatomical details [42]. Acquisition at different phases of the cardiac cycle optimizes vascular visualization, while the high temporal resolution captures the contrast dynamics. Three-dimensional reconstruction and processing techniques such as automatic lesion detection improve interpretation [43]. In the post-processing phase, different types of images are created, including axial, sagittal, coronal, and three-dimensional. Techniques such as maximum intensity projection, intensity curves, and perfusion techniques enhance the analysis. The combination of these images allows a complete assessment of pulmonary and vascular conditions and contributes to a more reliable diagnosis [44, 45].

In CTPA exams, images are stored in the DICOM (Digital Imaging and Communications in Medicine) format, the medical imaging standard developed specifically for medical images and offering advantages such as the inclusion of patient-related information, acquisition data, and other metadata required in the clinical context [46]. Other formats used in medical imaging research include the Neuroimaging Informatics Technology Initiative (NIfTI), Joint Photographic Experts Group (JPEG), Portable Network Graphics (PNG), Tagged Image File Format (TIFF), and Raw Image Format (RAW), each of which has specific properties. The PNG format is commonly used outside of medical practice; the TIFF format is uncompressed and suitable for medical details; and the RAW format contains raw data from CT scanners. [47, 48].

### Machine learning applied to medical imaging

Machine learning (ML), a subfield of AI, arises at the interface of computer science, statistics, and AI. ML enables AI systems to learn from previous experience, improve their performance, and perform complex tasks that would be difficult to program conventionally. This paradigm has implications for various fields, including pattern recognition, natural language processing, computer vision, medicine, and finance [49]. When applying ML to medical images, algorithms are used to analyze and extract information from images generated by diagnostic imaging devices. The goal is to train algorithms to recognize patterns, identify relevant features, and perform specific tasks, such as diagnosing diseases, segmenting anatomical structures, detecting anomalies, or classifying medical conditions.

ML in the medical context has the potential to support medical professionals by offering fast and accurate analysis tools, contributing to efficient and personalized diagnoses, and enabling the detection of patterns or features that are not immediately visible to the naked eye. Different neural network technologies are used to perform the ML task, such as convolutional neural networks (CNNs) and recurrent neural networks (RNNs). Both have common characteristics that make them complementary in different scenarios, although they belong to different categories of neural networks. They are designed to process complex data and have the ability to learn hierarchical representations. CNNs are specifically designed to process and analyze data for complex classification or segmentation patterns, such as medical images.

RNNs have internal loops so that previous information is retained and influences current predictions. They are designed to handle sequential data such as time series or natural language. Both architectures have their specific characteristics and are often combined or modified to meet the particular requirements of different ML tasks. CNNs are efficient for spatial data, while RNNs are efficient for sequential data. Newer architectures, such as convolutional recurrent neural networks (CRNNs), combine elements of both for tasks that require both spatial and sequential processing.

CRNNs represent a neural network architecture designed for tasks that require concurrent information processing. They are particularly useful in contexts in which the hierarchical structure of spatial features in data, such as images, needs to be captured together with temporally sequential information. Convolutional layers deal with the extraction of spatial patterns, while recurrent layers, which have internal loops, process sequential information while retaining long-term memory. Techniques such as InceptionResNet V_2_, a CNN architecture often chosen for computer vision tasks, and LSTM, an RNN architecture, can be used and integrated into broader machine learning models in which features extracted by LSTM are combined with other clinical data or radiological information.

#### Preprocessing in computed tomography images

Preprocessing of CT images involves a number of techniques aimed at improving quality, facilitating analysis, and extracting relevant information from the images, thereby extending their clinical utility [50]. Among the most important preprocessing methods for CT images, intensity normalization stands out, which corrects variations in pixel intensity and ensures data consistency. In addition, resampling is used to adjust the spacing between pixels or voxels to achieve an optimal spatial layout. The data augmentation technique creates artificial variations in the training data by introducing transformations and perturbations into the existing images to increase the diversity of the dataset. Manual segmentation, in turn, consists of the process of dividing the image into significant regions or structures, which involves the identification and extraction of relevant anatomical areas or imaging abnormalities. Finally, windowing adjusts the grayscale of the image through the use of Hounsfield units [HU] and allows the adjustment of brightness and contrast to highlight certain features. Varying the window size determines which HU are represented by different shades of gray, resulting in sharper and clearer images [51, 52].

## Methodology

**Fig 2:**
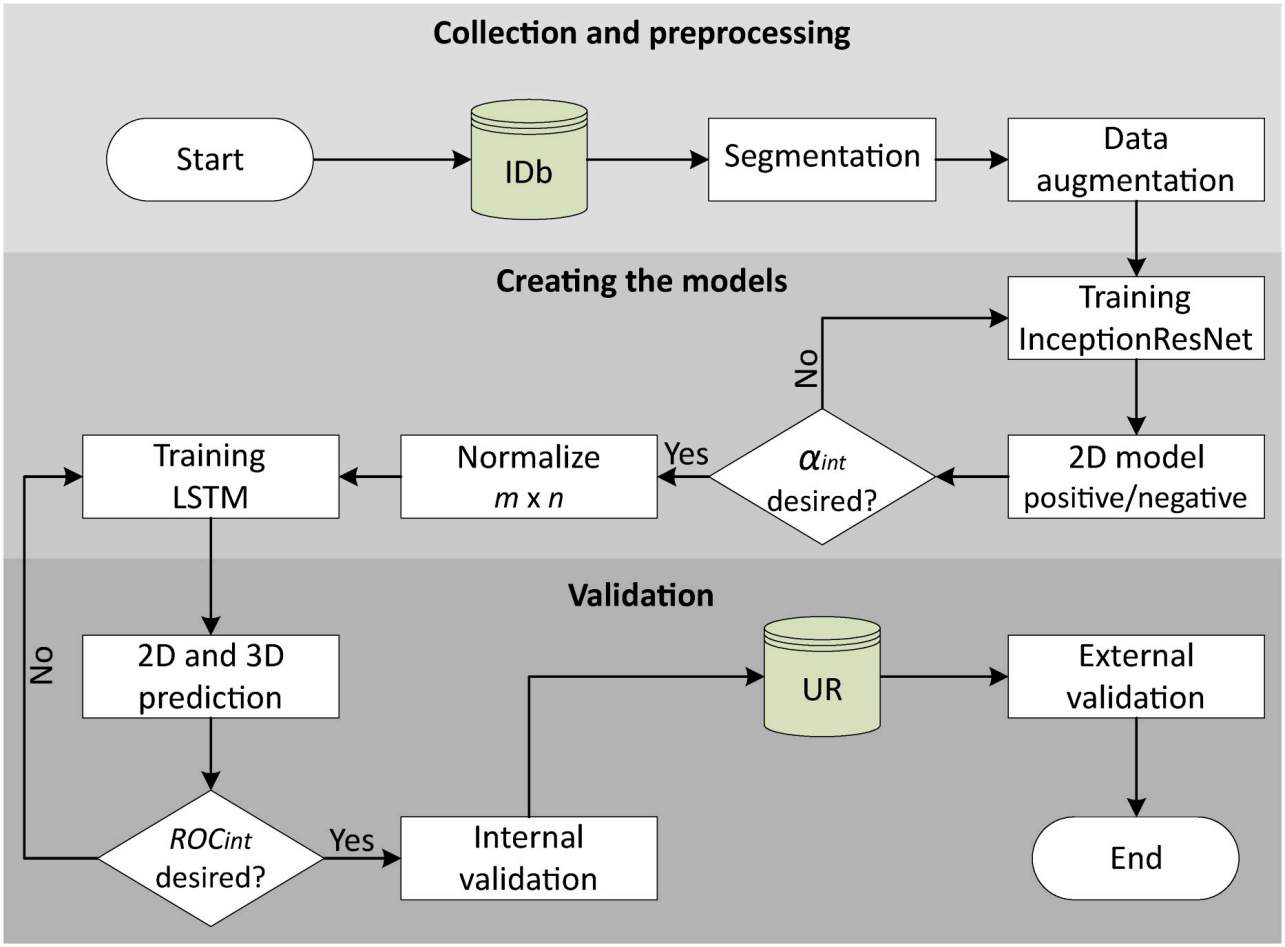
Flowchart of the proposed methodology.

The proposal for classifying PE in CTPA images adopts a two-dimensional approach that incorporates time series. Each scan slice is classified by the CRNN learning model, which in turn correlates the slices to generate predictions at both slice (2D) and scan (3D) levels indicating whether it is positive or negative for PE. The training of the CRNN model comprises two steps: i) CNN model InceptionResNet V_2_ and ii) RNN LSTM model. Both are trained with public datasets containing binary labels. Image segmentation, which is the division of an image into regions, objects, or pixels with the intent of separating objects of interest from other elements [53], isolates the lung region in the dataset and ensures that the model is only confronted with relevant examples. Data augmentation techniques are applied to expand the training set. First, the CNN is trained independently with 2D images. Then the RNN is trained for the 2D model without any data augmentation. The set of slices serves as input to the LSTM model and enables simultaneous predictions at 2D and 3D image level. The proposed workflow is shown in Fig 2.

### Data collection, analysis, and preprocessing

Initially, the CTPA exams classified as PE (both positive and negative) are collected in a public dataset to form the personalized subset of the database. These exams are stored in the Image Database (IDb). After storing the images in the IDb, segmentation is required, which can be manual or automatic. This process is performed to exclude regions from the exams that are not used for the PE application, such as the abdomen and neck. The goal of this process is to train the model using only the region of interest, which is the lungs. In this work, automatic segmentation is performed using image processing techniques that involve morphological operations.

When considering the 3D examination and defining the thickness of each slice, a greater number of PE-negative slices is observed compared to positive slices. The ratio between positive and negative slices is therefore unbalanced, with a higher number of negative slices. Consequently, data augmentation is performed to balance these numbers, including increasing the number of positive slices for the CNN training set. Data augmentation uses methods such as translation, rotation, blur, Gaussian noise, zoom, and elastic transformation. The exams are collected in DICOM format and later saved in the IDb in PNG format. The reason for this is the need for data compression to reduce the computational cost of training. All images stored in IDb and UR are anonymized.

### Creating the models

Then the training of the proposed models begins, starting with the CNN InceptionResNet V_2_. This architecture is trained to analyze 2D images and classify PE as positive or negative. Once the desired metric *α*_*int*_ is achieved, the exams undergo normalization, and the dimensions are adjusted in pixels. This normalization generates the encodings of the characteristics of each exam, which are extracted from the penultimate layer (dense layer) of the InceptionResNet V_2_ CNN model. The exams stored in the BDI are then divided into training and internal validation sets according to the concept of cross-validation.

The training of the InceptionResNet V_2_ CNN model aims to explore its advanced capabilities in discriminative feature extraction to perform accurate PE classification in CTPA images. The training of the RNN LSTM model, in turn, aims to deal with sequential data and capture temporal dependencies. This model enables the generation of predictions at slice and scan level. In addition, the LSTM RNN can model long-term relationships between image slices, enabling an understanding of the 3D image. Its ability to deal with sequences of different lengths is important in this context, especially considering that the number of slices can vary in 3D scanning.

### Internal and external validation

Following RNN-LSTM model training, predictions are generated at 2D and 3D image levels as shown in Fig 2. The performance of the model is evaluated both internally and externally. For internal validation, accuracy *α*_*int*_, loss *L*_*s*_, and receiver operating characteristic (ROC) *ROC*_*int*_ curves are considered, in addition to metrics such as recall *R*_*int*_, precision *P*_*int*_, *f*_1_-score $f_{1_{int}}$, and confusion matrix *C*_*int*_. For external validation, exams from the Universal Repository are used. The proposed model analyzes these exams and the results are compared with medical reports (which are considered the gold standard) using metrics such as precision *P*_*ext*_, recall *R*_*ext*_, confusion matrix *C*_*ext*_, *f*_1_-score $f_{1_{ext}}$, and ROC curve *ROC*_*ext*_. Detailed information on these metrics can be found at Chicco & Jurman [54].

## Results

This section presents the results obtained by applying the proposed method. In particular, the databases, the experiment design, the testing and configuration of the models, and their training performance are presented. The metrics used to evaluate the results are also discussed and a detailed analysis of the results for internal and external validation is performed.

### Public and internal dataset

The public PE database of the Radiological Society of North America (RSNA) was used for training and internal validation, with a dataset of 12,195 exams, totaling 2,995,147 images (https://www.rsna.org/rsnai/ai-image-challenge). From this dataset, a new set was created for the Kaggle platform recognition challenge [55], consisting of 9,446 exams, with a total of 1,790,594 images [56]. Three proprietary subsets were separated by the Kaggle Platform (https://www.kaggle.com/) for the challenge: 7,279 exams for the training set, 650 exams for the public test set, and 1,517 exams for the private test set [57]. The data subsets themselves consist of positive or negative exams for PE with slices that may or may not contain signs of PE. Of the subsets themselves, 2,368 exams are positive for PE and 4,911 exams are negative for PE. The slice thickness for each exam is 1.25*mm*, which allows for precise visualization of possible thrombi.

The model was validated externally with the Universal Repository database of medical images of the Hospital Israelita Albert Einstein (In Portuguese: https://bancodeimagens.io/), for which the institutional ethics committee has given its approval nº: 65628422.7.0000.0071. All images stored in IDb and UR have been anonymized. For the validation, 216 exams were randomly selected, of which 113 were negative and 103 positive, with a maximum of 300 slices each. The exams were collected from January 2007 to October 2020. Of the total number, 195 exams had a slice thickness of 1.00*mm*, and 21 exams had a slice thickness of 1.25*mm*. The devices used to perform the exams were Discovery NM 530c and Toshiba E7843 from General Electric Company Healthcare Ltd. Table 2 shows the characteristics of the data subset obtained from the Universal Repository, categorized by class.

**Table 2:** Characteristics of Universal Repository patients.

Condition	*μ* (*V*_*min*_ and *V*_*max*_)	Female	Male
Negative PE	44 (15-92)	91	22
Positive PE	58 (18-92)	68	35
Total	51 (15-92)	159	57

In Table 2, the values of mean *μ*, minimum *V*_*min*_, and maximum *V*_*max*_ of age, separated by sex, were used to evaluate the distribution in patients with positive and negative results for PE, which is useful for clinical and epidemiological analyzes related to PE. The *μ* age of the patients who tested negative for PE was 44 years (range 15-92 years); among them were 91 female and 22 male. The *μ* age of the patients who tested positive for PE was 58 years (range 18-92 years); among them were 68 female and 35 male. The total number of patients included in the analysis was 51 (age range 15-92 years); of these, 159 were female and 57 male.

### Computational experiment design

Randomly, 800 exams were collected from public RSNA data, of which 400 were positive and 400 negative for PE, with a maximum limit of 300 slices for each exam. The Python library pydicom was used to load the exams, totaling 181,747 slices, 16,492 positive and 165,255 negative, as shown in Table 3. For each examination, an automatic segmentation was performed to identify the lung region. In this region, windowing was performed with a window of 1,400 and a level of -600. Following segmentation, background removal operations, extraction of regions near the edge and morphological operations such as erosion, dilation, OTSU thresholding and closing were performed to generate the mask applied to the image, using Python’s OpenCV library. The original resolution of each slice was kept at 512 × 512 pixels. To highlight the region of interest for PE classification, a window with level = 100 and width = 700 was applied to each slice. Each image was converted from DICOM to PNG 8-bit.

**Table 3:** Dataset information.

Original data from RSNA	Amount of data
CTPA (stacks)	800
Positive	400
Negative	400
CTPA (slices)	181.747
Positive	16.492
Negative	165.255

**Fig 3:**
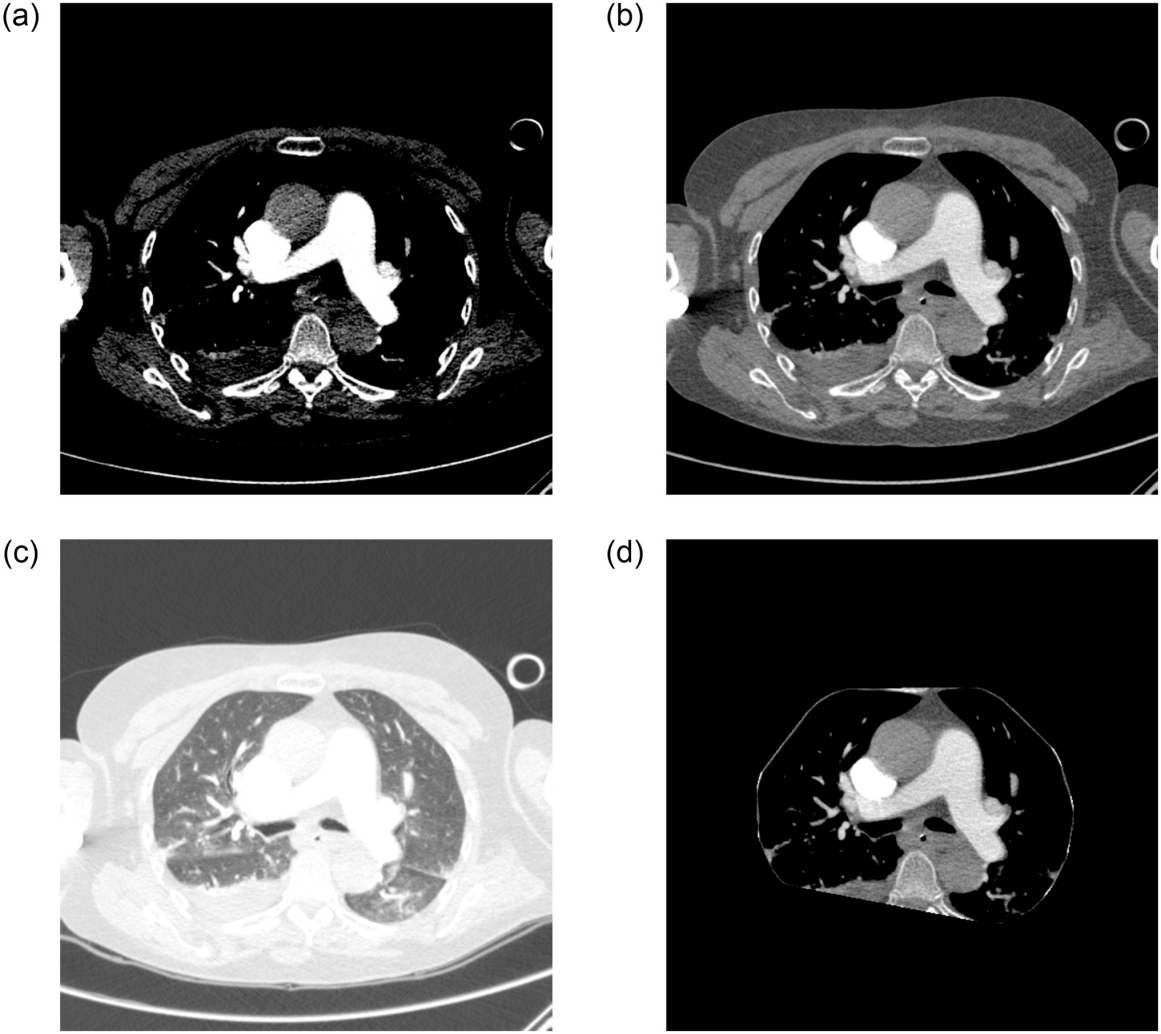
Application of automatic windowing and segmentation: (a) original image, (b) windowing for PE, (c) windowing for lungs, and (d) automatic segmentation.

Subsequently, the data was augmented by generating new images to improve the generalization of the InceptionResNet V_2_ CNN model. This was done by applying rotation, translation, and brightness changes, among others. Ten variations were created for each positive slice, totaling 164,920 images. The number of variations in data augmentation was determined by dividing the original number of negative slices by the number of positive slices. InceptionResNet V_2_ was trained to learn the features of positive or negative slices for PE. Only images in which the lung region was visible were considered valid, resulting in the exclusion of 50 positive slices. 330,125 slices were considered for training the 2D model, of which 164,870 were positive and 165,255 were negative. The encodings of the characteristics of each exam were extracted from the penultimate layer, the dense layer. Finally, the RNN LSTM model was trained to make predictions at the image and exam levels. Both techniques, CNN InceptionResNet V_2_ and RNN LSTM, were coded using the Keras/Tensorflow libraries. Fig 3 shows the application of windowing and the proposed automatic segmentation.

Therefore, the model using the CRNN classification approach was implemented. The 2D and 2D+LSTM models were trained using cross-validation with k = 5, for 25 and 50 epochs, respectively. An NVIDIA Tesla T4 16GB was used to train the model. The hyperparameters of the InceptionResNet V_2_ model were configured as follows: i) sigmoid activation function, ii) binary cross entropy loss (BCE), iii) Adam optimizer, iv) learning rate *η*_*adam*_ = 0.0005 and v) lot size *T*_*L*_ = 16. The hyperparameters of the LSTM model were defined as follows: i) sigmoid activation function, ii) binary cross entropy loss (BCE), iii) Nadam optimizer, iv) learning rate of *η*_*nadam*_ = 0.005 and v) batch size *T*_*L*_ = 16. The values of the parameters and hyperparameters were obtained through empirical method.

### Internal validation results

**Fig 4:**
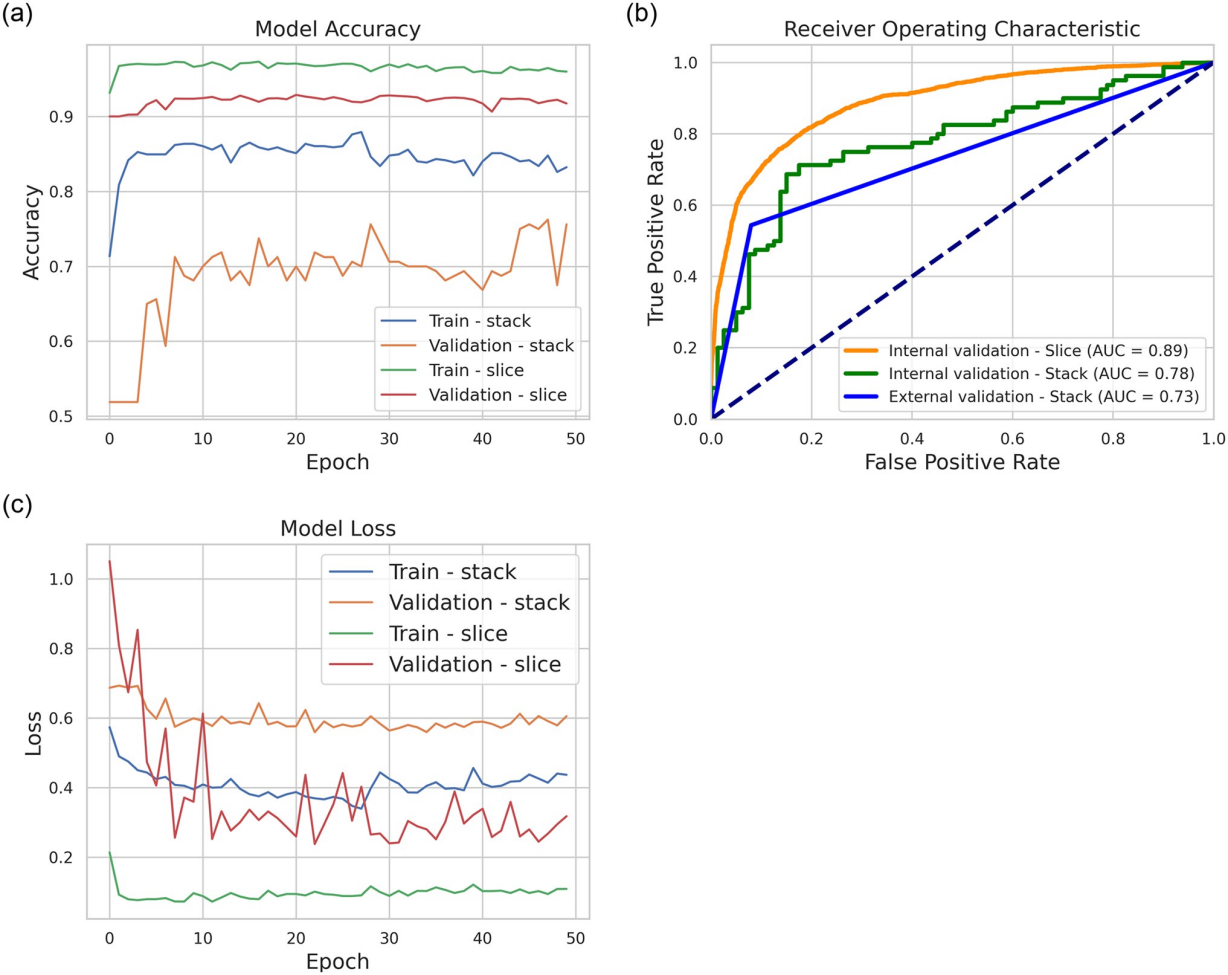
CNN-LSTM model metrics for PE classification at slice and stack levels: (a) precision curve, (b) ROC curve, and (c) loss.

For the exams used to train the model in this study, the reports are available at the exam (3D) and slice (2D) levels. However, the Universal Repository database only contains exam-level reports, without 2D analysis. Therefore, the model is validated externally at the exam level. The results for the classification model at the slice and exam levels are shown in Fig 4. Fig 4(a) shows that the accuracy value (*A*_*f*_) at the level of training slices was ≈97% (green curve), while the value of *A*_*f*_ for validation was ≈93% (red curve). The exam-level accuracy (*A*_*e*_) for training was ≈86% (blue curve), and the *A*_*e*_ for validation was ≈77% (orange curve). Fig 4(b) shows that the area between the model and the ROC curve for internal validation is 89% at the slice level and 78% at the exam level.

Fig 4(c) shows the cost function (loss), which decreases over the epochs. It varies between 0.2 and 0.5 in the training set and 0.5 to 0.3 in the validation set, each at the slice level. In Fig 4(c), it can also be seen that the loss in training and validation is larger at the exam level, indicating that it is difficult for the model to correlate features between slices of each exam. If the model were trained for more epochs, overfitting would occur.

Based on Fig 4(c), the training loss Train-stack, shown in blue, decreases and stabilizes, while the validation loss Validation-stack, shown in red, is higher and fluctuates without a clear increasing trend. The training loss Train-slice, shown in green, is low and stable, whereas the validation loss Validation-slice, shown in orange, is higher and more variable. For the stack model, the difference between training and validation losses does not increase significantly. In the slice model, the difference is larger, suggesting overfitting. If trained for more epochs, especially the slice model, the difference may increase, indicating that the model is memorizing patterns from the training data that do not generalize to the validation data. The low and stable training loss and the fluctuations in validation loss indicate overfitting.

### External validation results

Following the internal validation, an external validation was performed in the entire system at the exam level. Thus, considering the true positives *T*_*p*_, true negatives *T*_*n*_, false positives *F*_*p*_, and false negatives *F*_*n*_, the Table 4 presents the data from the confusion matrix, and the values of precision, recall, *f*_1_-score, and amount of data, divided by class for the external validation set, are shown in Table 5.

**Table 4:** Confusion matrices for internal validation.

Validation	*T*_*p*_	*T*_*n*_	*F*_*p*_	*F*_*n*_
Internal—slice	17627	1152	786	912
Internal—stack	66	55	14	25
External—stack	104	56	9	47

**Table 5:** Precision, recall and *f*_1_-score metrics for the internal validation set.

Class	Precision	Recall	*f*_1_-score	Amount of data
**slice level—internal validation**				
Negative PE	95%	96%	95%	18413
Positive PE	59%	56%	58%	2064
**stack level—internal validation**				
Negative PE	73%	82%	77%	80
Positive PE	80%	69%	74%	80
**stack level—external validation**				
Negative PE	69%	92%	79%	113
Positive PE	86%	54%	67%	103

**Fig 5:**
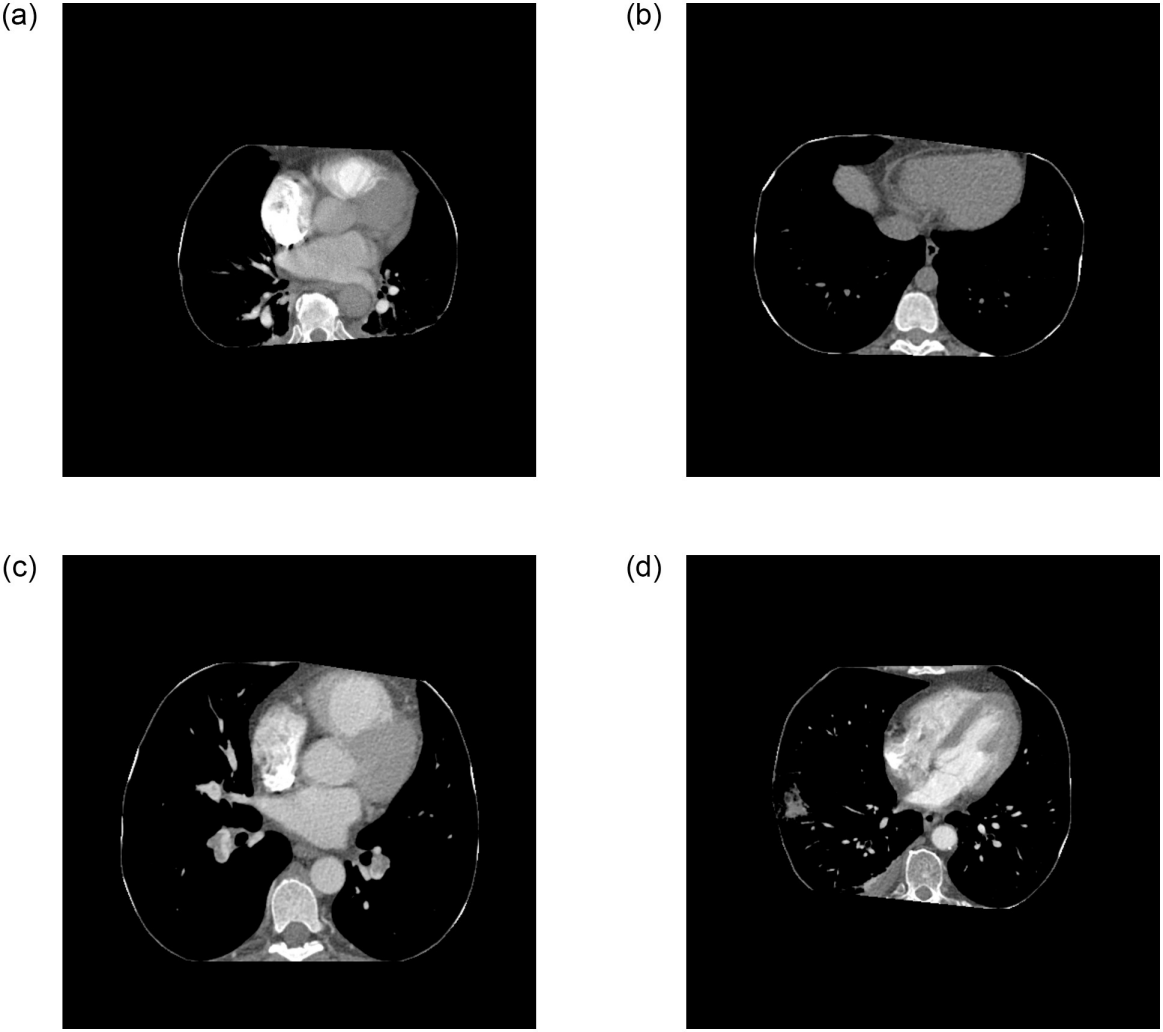
Classification of exams from the Universal Repository database: (a) *T*_*p*_, (b) *F*_*p*_, (c) *T*_*n*_, and (d) *F*_*n*_.

Of the 216 exams used, 195 had a slice thickness of 1.00*mm*, while the remaining 21 had a slice thickness of 1.25*mm*. This mixture of slice thicknesses demonstrates the ability of the model to generalize to exams with different specifications, indicating its potential for practical application in various medical scenarios. Fig 5 shows examples of *T*_*p*_, *T*_*n*_, *F*_*p*_, and *F*_*n*_ from exams classified by the model using the Universal Repository.

The accuracy (*A*_*f*_) of the model varies according to gender: 70.17% for *M* and 75.47% for *F*. The confidence intervals *ψ* for *A*_*f*_ in each age group, with a confidence level of 95%, are shown in Table 6, which also shows a tendency for *A*_*f*_ to decrease with increasing age. This trend is accompanied by a widening of the confidence intervals, indicating greater uncertainty in accuracy estimates for older groups. Sample sizes also vary, with the 40-60 age group being the largest and the 80+ group the smallest.

**Table 6:** Accuracy and confidence interval for age groups for the external database.

Groups	Amount	Accuracy	*ψ*
Less than 40 years old	68	83.82%	75.1% a 92.6%
Between 40 and 60 years old	81	79%	70.1% a 87.9%
Between 60 and 80 years old	49	67.34%	54.2% a 80.5%
Over 80 years old	18	33.33%	11.6% a 55.1%

**Fig 6:**
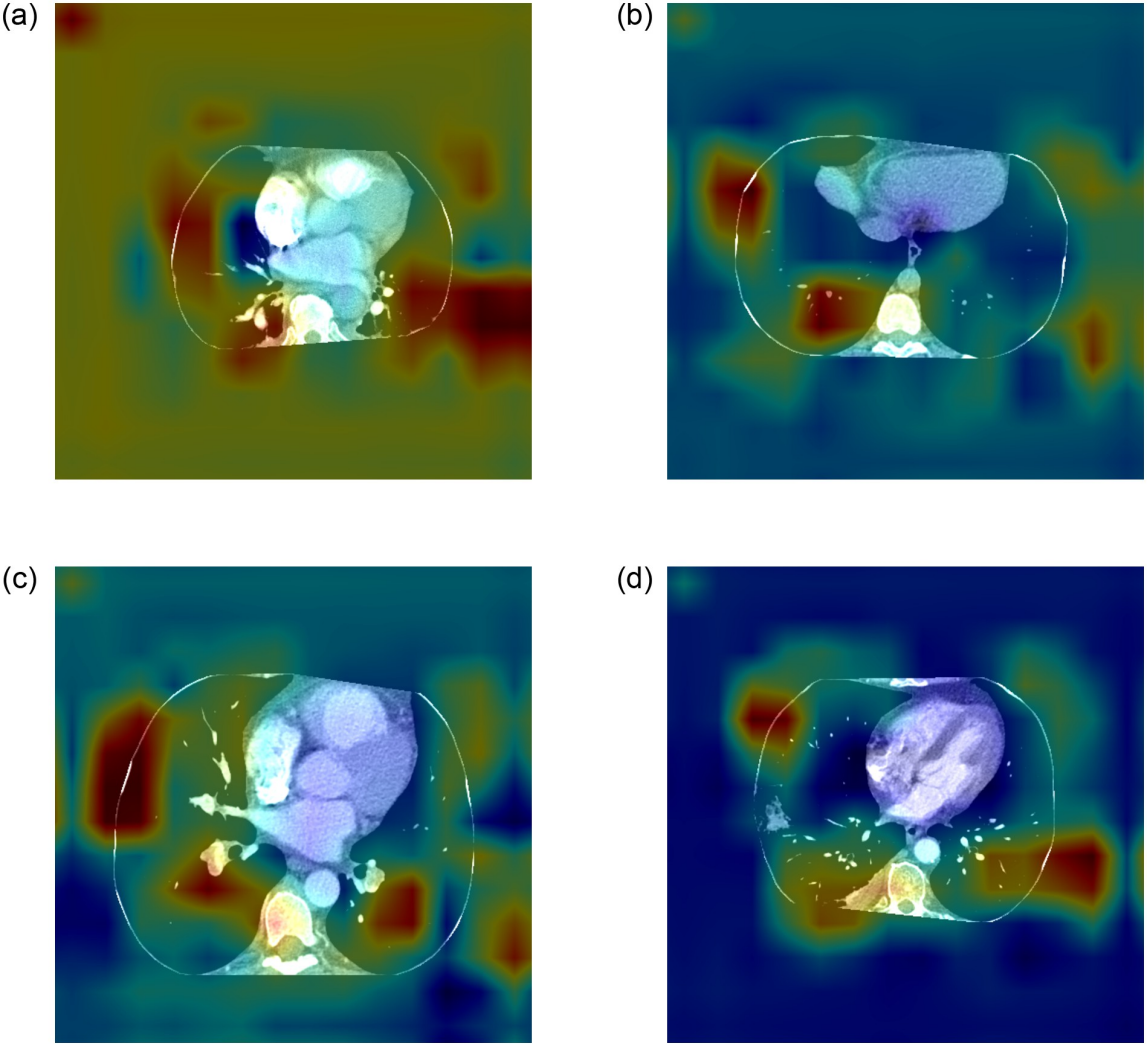
Universal Repository exam heatmaps: (a) *T*_*p*_, (b) *F*_*p*_, (c) *T*_*n*_ e (d) *F*_*n*_.

Fig 6 shows slice-level heatmaps for *T*_*p*_, *T*_*n*_, *F*_*p*_, and *F*_*n*_, with the same images as shown in Fig 5. The maps were generated from exams classified by the proposed model using the Universal Repository. These maps are often used to mark areas of varying density or intensity to help healthcare professionals identify abnormal features such as tumors, inflammation, or other pathological findings.

In Fig 6, cooler colors (from blue to green) indicate negative values or areas of lower intensity, concentration, or probability, while warmer tones (from red to yellow) indicate positive values or areas of higher intensity, concentration, or probability. Accordingly, *T*_*p*_ indicates regions where the model has correctly identified the presence of a particular feature, e.g. the accurate detection of an anomaly. *T*_*n*_ indicates regions where the model has not correctly identified the presence of the feature of interest, although it was not actually present. This shows that the model is working correctly when it discards regions that are irrelevant to abnormality condition. For *F*_*p*_, areas are shown where the model incorrectly indicated the presence of a feature that was not actually present. This could be due to noise in the data or to features that are similar to the abnormality condition. Finally, *F*_*n*_ indicates areas where the model failed to detect the presence of the feature of interest even though it was present in the data, which is problematic, especially when early detection of pathological conditions is required.

### Validation by trained radiologists

Two board-certified radiologists, $R_{p_{1}}$ (5 years of experience) and $R_{p_{2}}$ (10 years of experience), analyzed six exams without access to previous reports. The proposed model also classified these exams, with three classified as positive (P) and three as negative (N). Table 7 compared the classifications of these exams by the model, radiologists, and Universal Repository database reports. After the initial analysis, each radiologist received the processing data from the proposed model and integrated it into their new reports.

**Table 7:** Comparison between exam reports.

#	Proposal	$R_{p_{1}}$	$R_{p_{2}}$	UR
Exam 1	P	P	P	P
Exam 2	N	N	P	P
Exam 3	P	P	P	P
Exam 4	P	N	N	N
Exam 5	N	N	N	N
Exam 6	N	P	N	N

For $R_{p_{1}}$, the exams presented challenges in making the diagnosis, as CTPA normally has an average of 350 slices. According to $R_{p_{1}}$’s analysis, of the 128 slices evaluated in each exam, the areas of interest were located between slices 90 and 100, which led to the detection of about 30 slices with contrast-filling defects and increased the complexity of the procedure. In the case of Exam 3, $R_{p_{1}}$ observed a possible filling defect in the subsegmental branch of the right lower lobe. However, due to the limitation of the sections, which did not cover the entire area of the lung, it was not possible to confirm whether this branch was arterial.

$R_{p_{1}}$ emphasized that the PE classification model aims to detect contrast-filling defects in the pulmonary arterial system. Since only a limited number of slices are available, the radiologist’s judgment is impaired, which increases the likelihood of false-negative results. However, the proposed method allows evaluation even with a reduced number of slices. In addition, deficiencies were found in the anatomical segmentation of the lungs and in the heat maps, as the images did not cover the entire lung area, which reduces reliability for the user.

For $R_{p_{2}}$, the proposed detection model showed high agreement, but its application in image evaluation had some limitations. for $R_{p_{2}}$ highlighted that the analysis was performed on axial images covering only part of the lungs, which made reformatting in other planes and windowing impossible and limited radiologic evaluation. In clinical practice, analysis of the chest is performed using different window levels in multiple planes, in addition to considering the patient’s clinical data, laboratory data, and previous imaging exams. Another point of concern was the inclusion of extensive extrapulmonary segments in the heat maps, which had a negative impact on the ability to explain and interpret the model results.

$R_{p_{2}}$ described that despite the limitations, the significant reduction in the time required to produce results using the model stands out as an advantage over the time radiologists spend analyzing images. Considering that PE is a potentially fatal condition, applying an efficient detection model to recently performed CTPA exams can significantly reduce the time between image acquisition and final analysis by the radiologist. This process optimization leads to more efficient and optimized patient care.

Considering two radiology experts, the proposed model and the reports previously generated and stored in the UR, it was possible to measure the area under the receiver operating characteristic curve (AUC-ROC) obtained based on the exit probability scores and predefined by the reference standard, the Universal Repository reports. Sensitivity (*S*_*e*_), specificity (*E*_*e*_), positive predictive value (*V*_*PP*_), and negative predictive value (*V*_*PN*_) were calculated at the predefined operational cutoffs. The results of the performance analysis of the proposed model and the experts compared to the Universal Repository reports are presented in Table 8.

**Table 8:** Model and expert performance compared to reports.

	*A*_*f*_	*ψ* da *A*_*f*_	*V*_*PN*_	*V*_*PP*_	*S*_*e*_	*E*_*e*_
Proposal	≈ 66.7%	28.9% a 104.3%	≈ 66.7%	≈ 66.7%	≈ 66.7%	≈ 66.7%
*R*_1_	≈ 66.7%	28.9% a 104.3%	≈ 66.7%	≈ 66.7%	≈ 66.7%	≈ 66.7%
*R*_2_	100%	100%	100%	100%	100%	100%

### Discussion

Based on the results, the proposed classification model showed promising performance in detecting negative PE cases, with consistently high precision and recall at both slice and exam levels. The efficiency of the model in excluding false positive diagnoses suggests that it can be used as a reliable tool to correctly identify patients without PE, emphasizing its importance in clinical practice. However, the model’s performance in detecting positive PE cases needed improvement, as evidenced by comparatively lower precision and recall scores. Although the majority of relevant cases were identified by the model, there is still room for improvement in the detection of positive PE cases. The additional analysis of the externally validated model provided successful results indicating high accuracy for both classes. The high recall for the negative PE class indicates that the majority of patients without PE were correctly identified, while the considerable precision in the positive PE class indicates that the majority of patients diagnosed with PE actually had it. In addition, the model showed robustness and generalizability in dealing with different slice thicknesses in exams, an important property for applicability in various medical scenarios.

When considering demographic data, the patients with negative PE results were on average younger (53 years) than those with positive results (65 years). The sex distribution was also different: negative results had more female patients, while positive results had a more balanced distribution. By making specific adjustments to the model, such as hyperparameter adjustments, regularization, data augmentation, transfer learning, or fine-tuning, it is possible to increase precision and recall and thus improve the model’s ability to correctly diagnose PE.

The analysis shows that the proposed model and interpretation of radiologists $R_{p_{1}}$ and $R_{p_{2}}$ have different challenges and positive points. $R_{p_{1}}$ had difficulties due to the limited number of available slices, which affected the detection of contrast-filling defects in the pulmonary arterial branches. On the other hand, $R_{p_{2}}$ emphasized the high agreement of the model, but pointed out limitations in practical application, such as the limited analysis of the entire lungs. However, the model excelled in reducing the time needed to produce results, which is important for the early detection of diseases such as PE. These considerations show the importance of evaluating both the accuracy and practicality of the model.

The results obtained show a convergence with previous studies in terms of the ability to correctly classify negative and positive examples of PE and make predictions at 2D and 3D level [15, 19, 20]. Despite the lower performance compared to the metrics recorded in the literature, the validation of the model with a completely heterogeneous external database stands out, identifying characteristics of the problem such as the balance of the number of patients of both sexes, the inclusion of patients over 60 years of age and different slice thickness in exams.

From this study, an overview of the validation of AI models with a large and heterogeneous database comprising various medical devices was developed, filling a gap in the current literature. The main difficulty in developing the work was the lack of demographic information on the RSNA training database. The limitation of the work is related to the number of exams used from the Universal Repository database, both in terms of the number of patients of both sexes and the number of exams of different slice thicknesses. The main success relates to validation using metrics for age groups, gender, and overall accuracy.

As a continuation of this work, we intend to implement model explainability methods, such as GRADCAM and PCAM, which are of great importance in the medical field. Additionally, new modelings will be conducted based on recent advances in the literature, testing the latest available architectures. Other planned actions include the utilization of open datasets for retraining and validating the current model, as well as the implementation of Kaplan-Meier plots and Cox regression. To conduct these survival analyses, the collection of variables such as time to event, event status, and covariates will be necessary.

## Conclusion

This work aimed to develop a deep neural network capable of classifying PE from CT angiography images at the slice and volume level, using the Universal Repository of medical images for validation. A new CRNN architecture was implemented, added, and integrated, and data from different devices were used. The hypothesis of this study was confirmed by the results, as the model was trained with a public database and later validated with a large and heterogeneous database, minimizing the *F*_*p*_ errors. The objectives were achieved and enabled the integration of future CRNN architectures. The results of the external validation were satisfactory, showing good accuracy for both positive and negative classes. Notably, the high recall for the PE-negative class indicates accurate identification of most patients without PE, and the high precision for the PE-positive class indicates a correct diagnosis in most patients with PE. In addition, the model has demonstrated the ability to generalize across exams with different slice thicknesses, a crucial feature for real-world application. This leads to the conclusion that the model has practical applicability in various clinical settings.
